# Differential effect of anesthetics on mucociliary clearance in vivo in mice

**DOI:** 10.1038/s41598-021-84605-y

**Published:** 2021-03-01

**Authors:** Kyle S. Feldman, Eunwon Kim, Michael J. Czachowski, Yijen Wu, Cecilia W. Lo, Maliha Zahid

**Affiliations:** 1 Department of Developmental Biology, Rangos Research Center, University of Pittsburgh School of Medicine, 530 45th St., Pittsburgh, PA 15201 USA; 2 Diagnostic Imaging, Tomayko Group, Pittsburgh, PA USA

**Keywords:** Biotechnology, Physiology

## Abstract

Respiratory mucociliary clearance (MCC) is a key defense mechanism that functions to entrap and transport inhaled pollutants, particulates, and pathogens away from the lungs. Previous work has identified a number of anesthetics to have cilia depressive effects in vitro. Wild-type C57BL/6 J mice received intra-tracheal installation of ^99m^Tc-Sulfur colloid, and were imaged using a dual-modality SPECT/CT system at 0 and 6 h to measure baseline MCC (n = 8). Mice were challenged for one hour with inhalational 1.5% isoflurane, or intraperitoneal ketamine (100 mg/kg)/xylazine (20 mg/kg), ketamine (0.5 mg/kg)/dexmedetomidine (50 mg/kg), fentanyl (0.2 mg/kg)/1.5% isoflurane, propofol (120 mg/Kg), or fentanyl/midazolam/dexmedetomidine (0.025 mg/kg/2.5 mg/kg/0.25 mg/kg) prior to MCC assessment. The baseline MCC was 6.4%, and was significantly reduced to 3.7% (p = 0.04) and 3.0% (p = 0.01) by ketamine/xylazine and ketamine/dexmedetomidine challenge respectively. Importantly, combinations of drugs containing fentanyl, and propofol in isolation did not significantly depress MCC. Although no change in cilia length or percent ciliation was expected, we tried to correlate ex-vivo tracheal cilia ciliary beat frequency and cilia-generated flow velocities with MCC and found no correlation. Our results indicate that anesthetics containing ketamine (ketamine/xylazine and ketamine/dexmedetomidine) significantly depress MCC, while combinations containing fentanyl (fentanyl/isoflurane, fentanyl/midazolam/dexmedetomidine) and propofol do not. Our method for assessing MCC is reproducible and has utility for studying the effects of other drug combinations.

## Introduction

Cilia are microtubule based cellular organelles emanating from cell surfaces, that are evolutionarily conserved structures from algae to humans^1^. Cilia can be motile and multiple, functioning to generate directional flow, or single and non-motile, serving as cellular antennae to process sensory signals from the local environment^2,3^. Primary or sensory cilia are composed of nine peripheral microtubules, lacking the central doublet pair and dynein proteins, rich in receptors that act as a nexus for myriad key signaling pathways^2,4^. Motile cilia are multiple, with a few key exceptions like the embryonic node, beat in synchronized, metachronal waves to generate fluid flow such as in the lining of the tracheobronchial tree or fallopian tubes, or for providing cell motility as in flagellated sperm cells^1,2^. Motile cilia have the same nine peripheral microtubules with a central doublet and dynein arms which utilize the dynein motor proteins to move along the neighboring doublet in an ATP-dependent manner to induce a whip-like motion, a feature used for motility^1,5^.

The respiratory tract exposes an extensive epithelial surface to a variety of potentially hazardous pollutants and pathogens, such as dust, viruses and bacteria, inhaled with every breath. This necessitates a robust defense system to protect the respiratory tract from this constant onslaught. One key mechanism is the mucociliary apparatus of the tracheobronchial tree, which functions to entrap inhaled contaminants in a continuous flow of secreted mucus, and mechanically transport it out of the airway by ciliary action from muti-ciliated apical cells lining the tracheobronchial tree that beat in metachronal waves transporting mucus and trapped particles cephalad, a feature essential for the normal functioning of the mucociliary clearance (MCC) apparatus^5,6^. This key role is amply demonstrated in patients suffering from primary ciliary dyskinesia (PCD), a congenital disorder marked by mutations in key ciliary proteins leading to immotile or poorly functioning respiratory cilia, which in turn leads to recurrent lung infections and bronchiectasis. Patients may in some cases have situs inversus totalis, also known as Kartagener’s syndrome^7^. It has been increasingly recognized that motile cilia at the node of the developing embryo break the left–right symmetry and specify left–right patterning in the developing embryo. Evidence for this is the number of mutations in cilia related genes identified in congenital heart disease (CHD), as the heart is one of the most asymmetric organs in the body^8^. Further work into ciliary function in patients with CHD revealed a high incidence of ciliary dysfunction in their respiratory tracts leading to increased prevalence of chronic upper and lower respiratory tract symptoms, as well as increased post-operative respiratory complications^9–12^. Additionally, CHD patients with ciliary dysfunction had increased risk for post-operative respiratory complications^5,10,12^.

The effect of anesthetics on ciliary function has been a subject extensively studied for many decades, albeit under in vitro culture conditions. Inhaled anesthetics such as isoflurane, halothane, and sevoflurane have been shown to significantly decrease ciliary beat frequency (CBF)^13–15^. Additionally, many common intravenous anesthetics and analgesics such as dexmedetomidine, midazolam, propofol, and lidocaine are also known to adversely affect CBF^13,^^16–18^. Our previous work using mouse tracheal epithelial cells, or human nasal epithelial cells ex vivo, also demonstrated dexmedetomidine and isoflurane as having cilia depressive effects, while fentanyl produced cilia stimulatory effect^13,19^. The combination of dexmedetomidine and fentanyl canceled each other out and produced no significant effect, as studied in excised mouse trachea epithelia^13^. All these studies on cilia function, as well as our own, were conducted on tracheal epithelial cells grown in an air–liquid interface or carried out on excised mouse tracheas. Given these reports in the literature of differential effects of anesthetics on ciliary motility in vitro, we hypothesized that these anesthetics will affect mucociliary clearance in vivo to varying extent given their differential effects on cilia motility. In this era of increasing environmental pollution, rising incidence of chronic lung conditions like chronic obstructive pulmonary disease, novel pathogens, and increased ventilator usage that require anesthetics, an in vivo study of common anesthetics would be of significant clinical relevance in identifying drugs that do not impair ciliary motility. In the current study, we sought to investigate the effects of various inhaled and intravenous anesthetics on MCC in vivo in mice. Our findings provide insight into the effects of commonly used anesthetics in the context of an in vivo system, which could be deployed for the selection of anesthetic and analgesic combinations that optimize ciliary function, reduce post-operative respiratory morbidity, and ultimately improve patient outcomes. A study by Hua and colleagues reported a differential effect of anesthetics on nasal, but not lung, mucociliary clearance with pentobarbital and avertin having more significant depressive effects on clearance compared with isoflurane^20^. However, to the best of our knowledge, this is the first study of its kind evaluating effects of anesthetics on MCC in vivo in mice. 

## Results

### Effect of anesthetic on mucociliary clearance

In order to rule out confounding from different central to peripheral deposition patterns, we calculated the central-to-peripheral (C:P) ratios for each time 0 scan and compared the anesthetics challenged mice to their baseline values. We found no significant difference in the pattern of C:P distribution (see Supplemental Table S1). The baseline MCC was 6.4 ± 2.8 The isotope deposition through intratracheal deposition followed by aspiration was effective, producing a uniform distribution of isotope in the lungs (Fig. 4). Isoflurane treatment reduced the clearance to 3.7 ± 3.0%, which did not reach statistical significance (p-value = 0.12) (Fig. 5A). The ketamine/xylazine treatment produced mean MCC values of 3.7 ± 1.3%, which was significantly lower than the baseline (p-value = 0.04; Fig. 5B). The dexmedetomidine/ketamine treatment reduced MCC significantly to 3.0 ± 3.4% (p-value = 0.01) (Fig. 5C). The fentanyl/isoflurane treatment produced a lung clearance of 5.8 ± 4.2%, which was not significantly different compared to baseline values (p-value = 0.40; Fig. 5D). Propofol challenge in isolation also did not decrease MCC significantly with a baseline value of 5.1 ± 2.4% decreasing to 4.5 ± 3.4% (p = ns) (Fig. 5E). Additionally, a combination of fentanyl, dexmedetomidine and midazolam also did not change MCC significantly from baseline with a baseline of 5.1 ± 2.4% changing to 5.2 ± 3.6% (p = ns) (Fig. 5F).

### Correlation of cilia characteristics with MCC scans

Based on initial study results, the first 5 mice enrolled into the study underwent pre-terminal repeat MCC scans, followed by euthanasia, tracheal harvest and cilia characteristic measurements. The cilia length in our wild-type B6 mice was 3.5 ± 0.60 microns, with a mean CBF of 5.9 ± 1.0 Hz. The measured percent ciliation was 23.2 ± 5.3%, with a measured cilia generated flow of 7.8 ± 1.6 μm/s. These results did not correlate with the pre-terminal MCC values. None of the in vitro cilia measurements correlated with the pre-terminal MCC scan, indicating that no single in vitro cilia attribute captures the complex in vivo MCC process.

## Discussion

Motile cilia lining the tracheobronchial tree are a first line of defense against inhaled pathogens and pollutants. In this era of novel viral pathogens, multi-drug resistant bacterial pathogens, and ever-increasing environmental pollution, their importance cannot be over-stated^5,21^. Motile cilia are exquisitely sensitive to myriad insults, including not only pathogens and pollutants, but also drugs in common clinical use^13,19,22^. As patients intubated and on mechanical ventilation in the intensive care units, or even those undergoing surgery, need to be sedated, the choice of drugs becomes an important decision. With this background in mind, we believe our study is a valuable addition to the existing literature.

There are several important findings. Our study shows that there is a large variation in MCC in mice, even in those from the same in-bred strain. We have observed large variations in MCC with clearances as high as 14.1% in C57BL/6 J mice (Supplemental Figure S1). However, repeated measurements of the individual mice have produced consistent clearance values, which supports the reproducibility of our method^23^. The variations in clearance are likely to be exaggerated between mouse strains due to key biological differences. For example, C57BL/6 mice are known to spontaneously develop hydrocephalus, which could be due to dysfunction of the motile cilia lining the ventricles^24^. A comparison of lung structure between C57BL/6, A/J, and BALB/c mouse strains indicated that there are significant differences in airway structure between strains^25^. Additionally, a comparison of lung repair following acute bronchial injury in C57BL/6, 129/TerSv, and 129/SvEv mouse strains showed significant strain related differences in repair progress^26^. Therefore, factors like mouse strain and sex while comparing effect of drugs like anesthetics, or interventions is key and needs to be kept constant so as not to introduce confounding in the results.

Our study revealed clear differential effects of anesthetic agents on MCC in vivo in mice. Ketamine based anesthetic combinations led to a significant decrease in MCC, with isoflurane alone showing a decrease in MCC that did not meet statistical significance due to large standard deviations in the data. However, in our experience, if mice were kept sedated constantly using isoflurane between the 0 and 6 h timepoints, the clearance was essentially zero (data not shown). The most notable result in this study is the lack of MCC depression when using fentanyl/isoflurane, or propofol alone, unlike the other anesthetic combinations. In fact, the data suggests that using fentanyl in combination with isoflurane or dexmedetomidine/midazolam abrogated the decrease in MCC values with no significant change in these values from baseline. Although it would be interesting to assess the effects of fentanyl alone on MCC values, we could not achieve this as the degree of anesthesia achieved with fentanyl alone was not adequate to allow for these mice to be restrained and hold still for the duration of the dual-modality SPECT/CT scan, necessitating use of other drugs in combination with it. Similar to our findings, bronchial mucus transport velocity was shown to be reduced in patients receiving sevoflurane and remifentanil combination in contrast to a propofol and remifentanil combination^27^.

Our findings are in line with our previous studies that utilized in vitro methods in mouse and human cells^13,19^. Inhaled anesthetics such as isoflurane have been shown to significantly impair airway ciliary function^14,15^. The same is true for intravenous agents including dexmedetomidine and ketamine, whereas fentanyl has been shown to have no effect on airway function^16–18^. All of these studies tested different anesthetic agents alone, or in combination, using human nasal ciliated cells, or mouse tracheal cells in vitro with or without growing these cells in an air–liquid interface. To the best of our knowledge, this differential effect of anesthetic agents on in vivo MCC in mice using the radio-isotope clearance method is the first of its kind. The in vivo method of measuring MCC used in this study generated data that was both consistent with previous in vitro data, and of greater biological relevance^13^. It provides a robust method for assessing the effects of anesthetics on airway cilia motility and resulting clearance in vivo. It should be noted that no single ciliary parameter ex vivo (percent ciliation, ciliary beat frequency or flow assessed with beads) had a significant correlation with MCC indicating that the latter is a complex process not adequately recapitulated by any single ex vivo cilia parameter.

The choice of sedatives has clinical significance in the post-operative period and beyond. Should our findings be confirmed by others, it would suggest that patients would benefit from a choice of anesthetics, in the surgical or intensive care unit, that keeps ciliary health and MCC clearances in mind, in an attempt to minimize post-operative or ventilator associated respiratory complications. Furthermore, we have outlined a method for in vivo MCC measurements that can be utilized by investigators to assess the effect of additional drugs, like beta blockers, nitric oxide donors, vasopressors etc. commonly used in the intensive care unit, on MCC.

### Limitations

Our study has several limitations. Although we confirmed the validity of our MCC method by measuring clearances repeatedly in a cohort of mice, our study size was small. It was not geared or powered to identify sex-associated differences in MCC, which would indeed be an area of relevance to investigate in subsequent studies. In terms of drugs tested, not all possible anesthetics or combinations, such as midazolam alone or fentanyl alone were studied, and hence no conclusions regarding their stand-alone effect on MCC can be reached. Further studies, with a larger number of drugs in current clinical use, need to be tested to assess the effects of these drugs on MCC. Nevertheless, our study provides a reproducible methodology that can be applied to test myriad variables/drug combinations of clinical relevance.

## Conclusions

Our study points to a differential effect of anesthetics on MCC. Specifically, unlike the other drugs tested, alone or in combination, fentanyl did not depress MCC in vivo*,* consistent with previous in vitro and ex vivo data*.* Additionally, propofol alone did not depress MCC. In contrast, ketamine containing combinations, with dexmedetomidine or xylazine, did depress MCC statistically significantly. Isoflurane alone did decrease MCC from a mean of 7.2 to 3.7% but this difference did not reach statistical significance and was abrogated by use of isoflurane in combination with fentanyl. Importantly, none of the ex vivo cilia characteristics correlated with MCC values, indicating that the latter is a complex process not predicted by a single cilia variable. Lastly, our method of assessing MCC in vivo is a robust one, and can be utilized to assess effect of multiple pathogens, environmental insults, or drugs on mucociliary clearance.

## Methods

### Baseline mucociliary clearance

**Figure 1:**
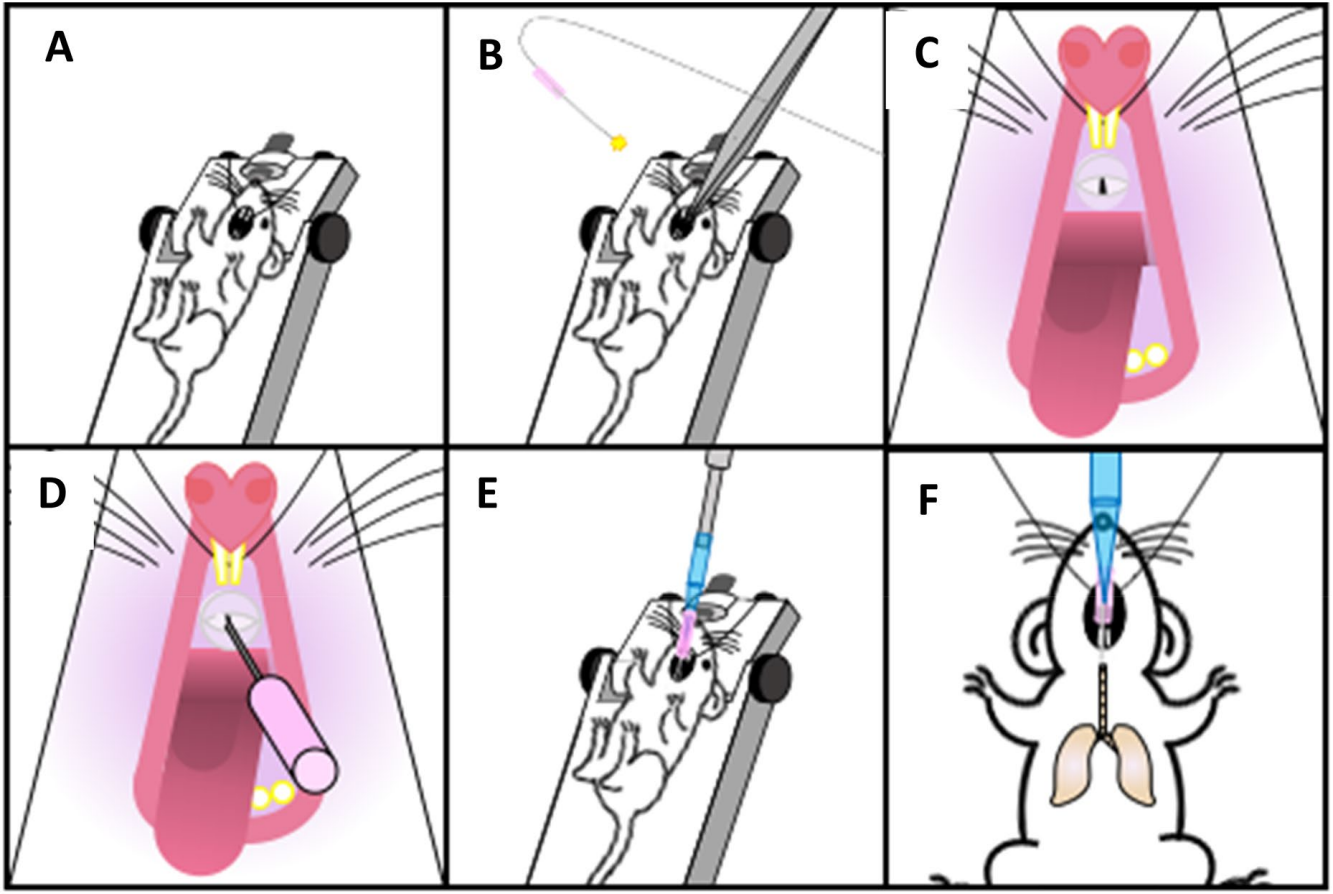
Mouse intubation and isotope instillation. Schemata displaying the steps for tracheal intubation and instillation of radioisotope. (**A**) Anesthetized mouse is placed onto a vertical support, suspended by the front incisors. (**B**,**C**) Mouth of the mouse is opened using forceps and a 20G cannula with an illuminated 0.5 mm fiber optic wire as a guide wire is prepared. (**D**) The mouse is intubated and (**E**) ^99m^Tc-Sulfur colloid is instilled into the cannula using a pipette, and (**F**) the mouse allowed to spontaneously inhale the isotope into the lungs.

All animal protocols were approved by University of Pittsburgh’s Institutional Animal Care and Use Committee. All methods were performed in accordance with institutional guidelines in the care of use of animals in research consistent with the ARRIVE guidelines (http://www.nc3rs.org.uk/page.asp?id=1357). For the study we utilized wild-type 6-week old C57BL/6 J mice (~ 25 g). A gap of at least 8 half-lives for technetium sulfur colloid (^99m^Tc-Sc) was given between testing various agents to allow for complete isotope decay. Baseline MCC scans were defined as MCC scans acquired without pre-challenging the mice with 1-h of sedation with various anesthetics. Baseline MCC scans were acquired by anesthetizing mice with 2% isoflurane (Henry Schein; 118–2097) for ~ 30 mins, and placing onto a vertical intubation tray (Kent Scientific; ETI-MSE-01) suspended by the front incisors (Fig. 1A). The support was adjusted to a 45° incline, and mouse intubated with a 20G cannula (Fisher Scientific; NC1534477), using an illuminated 0.5 mm fiber optic wire (Edmund Optics; 02-532) as guide and illumination source (Fig. 1B–D)^28^. Approximately 0.2 mCi of ^99m^Tc-Sc in 10μL of volume was instilled into the cannula resting in the trachea using a pipette, and mice were allowed to spontaneously aspirate it into the lungs (Fig. 1E,F). Following inhalation, mice were placed in an Inveon dual-modality SPECT/CT system (Siemens Healthineers, Erlangen, Germany) and a time 0 image acquired with 5-MWB-1.0 collimators, while maintaining sedation during image acquisition with 1.5% inhalational isoflurane through a nose cone. For ease of co-localization of the SPECT and CT images, a radioactive phantom was created by placing a dose of 0.05 mCi in 200 μL in a 0.2 mL PCR tube positioned on the mouse’s lower abdomen, far enough from the lung fields to prevent overlap or shadow artifacts. After this imaging, mice were allowed to recover and move around in cages freely with ad libitum access to food and water. Six hours later, mice were re-anesthetized with 1.5% isoflurane and imaging repeated. For evaluation of effect of various anesthetic combinations, mice were anesthetized for a total of 90 mins with only the anesthetic(s) of choice prior to time 0 image acquisition. The 6 h time-point image was acquired using inhalational 1.5% isoflurane.

In order to rule out any confounding from unequal deposition of technetium in the lungs, all scans reported in this study were analyzed for central-to-peripheral (C:P) radio-isotope deposition. The time 0 MCC scan images were analyzed for distribution within the right lung by quantifying the C:P ratio. First, the borders of the right lung were traced based on the CT scan images, followed by a nine-box grid was superimposed. The upper middle box of the grid was categorized as central lung and the remaining boxes were designated as lung periphery. The central to peripheral (C:P) ratio was calculated by dividing count density in the central region by count density in the peripheral lung^29^.

The SPECT images were acquired using a 40 cm radius of rotation, 60 projections with a 6° angle between projections, for an acquisition time of ~ 12 mins. CT images were acquired using 220 projections, a 1.6° angle between projections, for an acquisition time of ~ 4.5 mins. At the end of imaging, mice were recovered, allowed to move freely in their cages with access to food and water ad libitum. A second set of images similar to the first were acquired 6 h later in order to calculate MCC over this time-period. SPECT images were histogrammed and reconstructed to generate images of 60,000*60,000*94,500 microns with a scale of 0.002 pixels/micron. The CT images were reconstructed to generate images of 44,206.85*44,206.85*41,712.54 microns with a scale of 0.0261 pixels/micron. MCC analysis was performed using FIJI software^30^. All Measurements were taken by co-localizing the CT and SPECT images and selecting the right lung as the region of interest (ROI) based on a stack summed mask of the right lung. The right lung was chosen to avoid any overlapping signal from radioactivity potentially swallowed and present in the stomach, and quantified by summing the number of counts present in every image (counts per pixel unit). MCC was calculated as the percent difference in radiation counts between the 0 and 6 h time-points, with the 6 h counts in the ROI decay corrected using the formula: ${N\left(t\right)=N}_{0}{e}^{-t}$. ^99m^Tc-Sc has a decay constant of 3.21 ${e}^{-5}$ per second with a half-life of ~ 6 h^23^.

### Evaluation of anesthetics on mucociliary clearance

**Figure 2:**
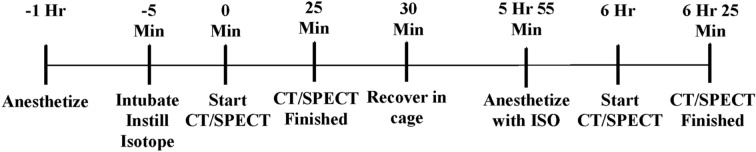
Anesthetic pretreatment and MCC measurement timeline. A timeline showing the 1 h anesthetic pre-treatment period, intubation time, scan time, post-scan recovery time, and the post 6 h re-scan time. This timeline was applied to every mouse and every anesthetic treatment in this study.

Evaluation of effects of various anesthetics on MCC was carried out by pre-treating mice with the anesthetic or anesthetic combination for 1 h prior to the 0 h image for measuring MCC, as detailed above. The dose for each anesthetic was determined by prior experimentation on mice to allow sedation to last for ~ 90 mins, the time needed for both the treatment and the 0 h time-point scan (Fig. 2). The anesthetics tested were; 1.5% isoflurane (Henry Schein; 118–2097), 100 mg/kg ketamine (Covetrus; 010177) with 20 mg/kg xylazine (Covetrus; 033197), 0.5 mg/kg ketamine with 50 mg/kg dexmedetomidine (Covetrus; 034362), 0.2 mg/kg fentanyl (Covetrus; 055012) with 1.5% isoflurane, 120 mg/kg propofol, 0.025 mg/kg fentanyl with 2.5 mg/kg midazolam and 0.25 mg/kg dexmedetomidine. All drugs were administered intra-peritoneally except for isoflurane which was administered via the inhalational route. All doses were selected to be of sufficient and equivalent depth so that the mice did not respond to a toe pinch. The 6 h scan was conducted by re-anesthetizing the mice with 1.5% isoflurane, matching the previous section (Fig. 2). Testing of various agents was separated by at least a gap of 8 half-lives to allow for complete decay of ^99m^Tc-Sc.

### Mouse tracheal harvest and airway analysis

**Figure 3:**
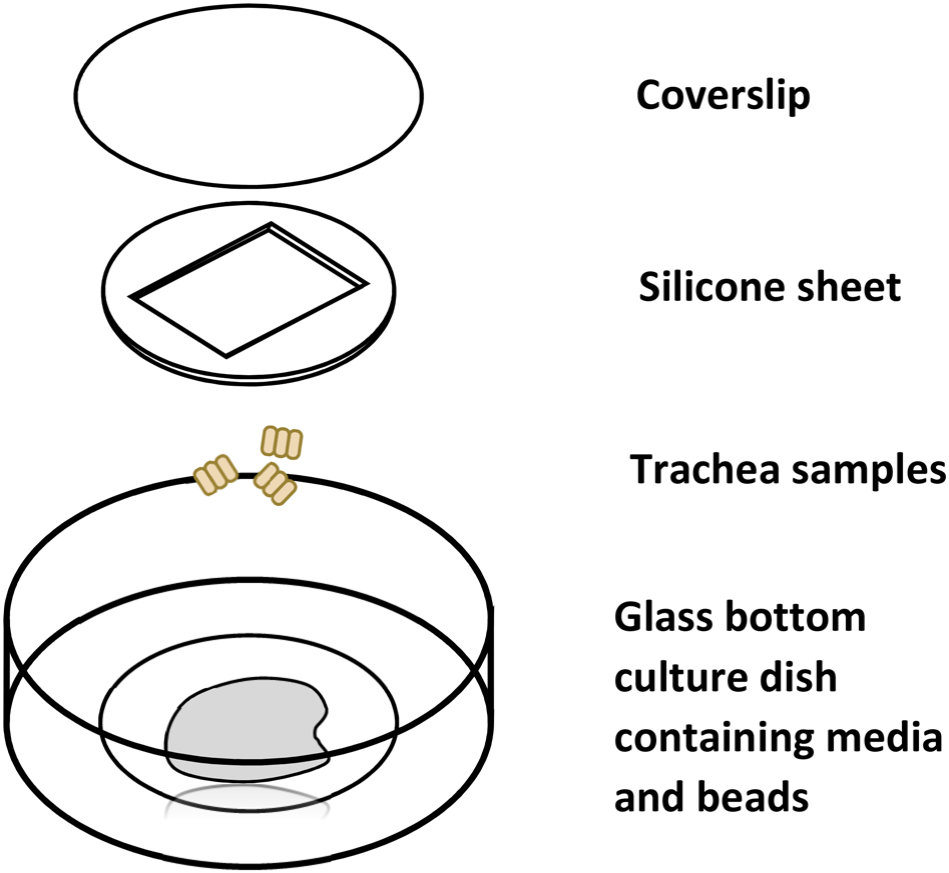
Culture dish set up and trachea sample positioning. Schemata illustrating the imaging setup of harvested tracheas. The silicon sheet is place into the cell culture dish, followed by media, then the trachea sample, and held in place with a coverslip.

**Figure 4:**
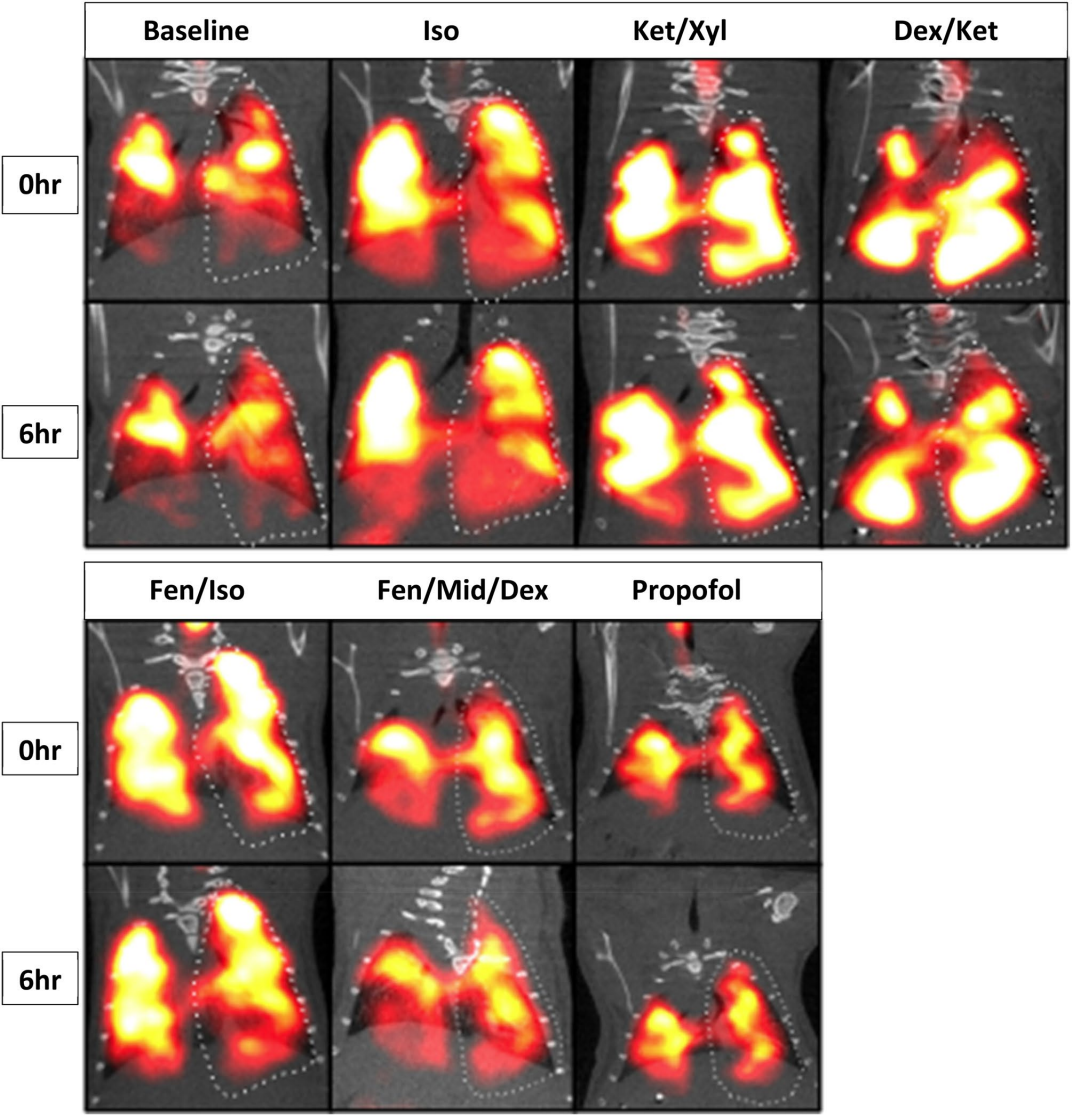
CT/SPECT colocalized images of MCC. A representative animal from each anesthetic group is displayed. The 6 h images are decay corrected and all of the image pairs for each anesthetic have matching image thresholds for visual comparison.

At the conclusion of the study, mice were euthanized using a high-flow CO_2_ inhalation in accordance with recommendations of the Institutional Animal Care and Use Committee. Tracheas were harvested, placed into culture dishes containing Leibovitz-15 media, cleaned of excess tissue, and splayed open lengthwise. The splayed tracheas were cut into squares, and transferred lumen side down to a 35 mm dish with 20 mm optical glass bottom. To the media bathing the tracheal pieces, 0.20 μm microspheres were added (16μL of beads from a 35μL of stock solution added to 500μL of Leibovitz-15 media) to give enough beads in the 100 × high-power field to assess flow generated by beating cilia. A 0.3 mm silicone sheet with an ~ 10 mm square trimmed in the center was placed around the trachea topped by a glass cover slip (Fig. 3)^31^. The sample was placed under an inverted microscope where cilia motion was captured as videos using a Leica DMI3000 B 100 × differential interference contrast oil objective (Leica Microsystems, Wetzlar, Germany)^31^, at 200 frames/s with a Phantom v4.2 camera (Vision Research, Wayne, NJ, USA). A minimum of 5 videos were acquired per trachea, with a minimum length of 1000 frames per video for analysis. Videos were analyzed for cilia length, CBF, percent ciliation and ciliary flow using FIJI software^30^ (see supplemental method S1 for details). Cilia length was measured by generating a binary mask of the cilia based on motion using an image processing macro (Supplemental Macro S1). These masks provide an easy measurable shape that matches the length of the cilia using a macro in ImageJ designed to measure the thickness of binary objects (Supplemental Macro S2). CBF was calculated by selecting areas containing either single cilia or groups of cilia beating synchronously and quantifying the difference between every image in the video compared to the first frame (Supplemental Macros S3, S4). These values were graphed and number of frames between peaks measured to determine CBF. Percent ciliation was measured for each image of the airway by measuring the total length of the tissue and the length of ciliated tissue. Percent ciliation was calculated using the ratio of ciliated to total surface. Cilia flow was measured by first processing the image to improve bead contrast (Supplemental Macro S5), followed by a feature point tracking algorithm that can be used to automatically track and quantify particle trajectories in videos. The tracker was used to output x and y coordinates for each particle^32^. Particle tracks within the range 50–300 frames were analyzed, while tracks outside of that range were excluded from the analysis, to avoid noise or Brownian motion artifacts (Fig. 4).

### Statistical analyses

**Figure 5:**
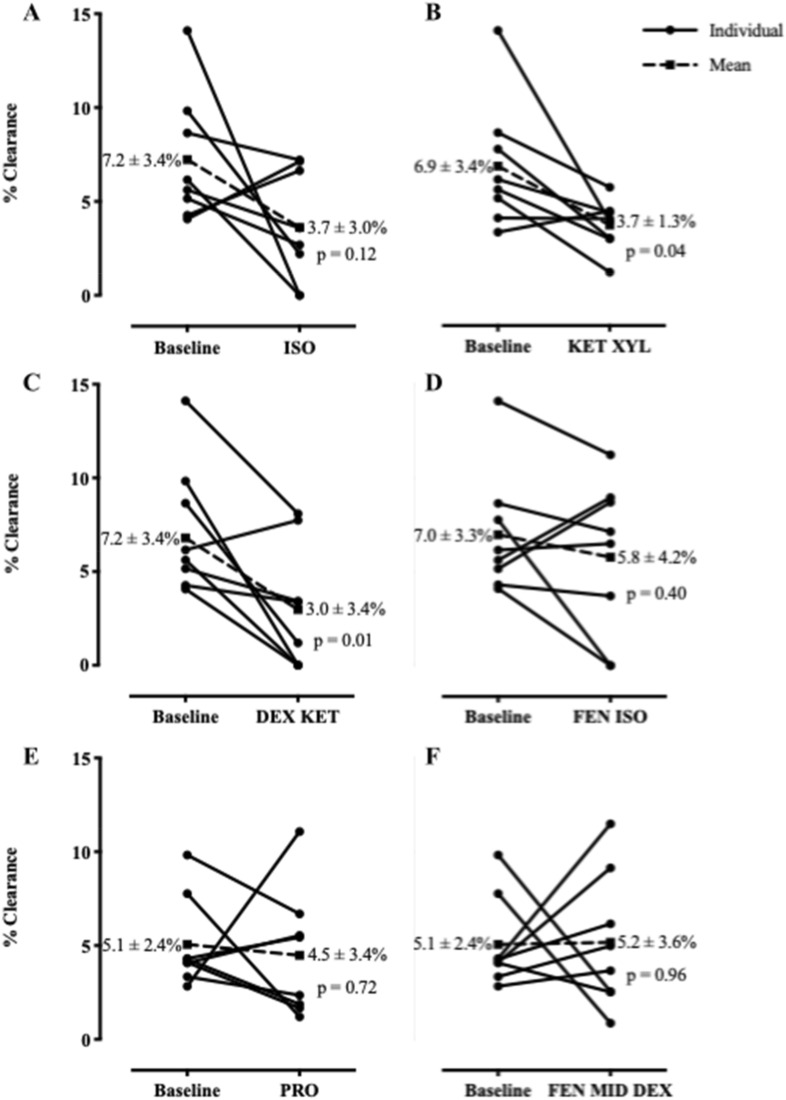
Measured MCC for each anesthetic. Individual clearances for each mouse are graphed in before-after line graphs. Each graph shows the baseline clearance and the clearances after anesthetic treatment. Mean clearances ± SD, and respective paired t-test p-values are graphed comparing each anesthetic to the baseline. (**A**) Isoflurane. (**B**) Ketamine and xylazine. (**C**) Dexmedetomidine and ketamine. (**D**) Fentanyl and isoflurane. (**E**) Propofol. (**F**) Fentanyl, midazolam, and dexmedetomidine.

Our preliminary data had showed a baseline clearance of 7.9 ± 1.7%. Assuming a power of 85% to detect a decrease in clearance of at least 33% to be meaningful with an alpha error of 5% or less, we reached a sample size of 8, which was subsequently used for making the baseline measurements as well as testing various anesthetics. MCC clearances are presented as mean ± SD of percent clearance at 6 h. Airway analysis performed for each mouse is presented with average cilia length in microns, average CBF in hertz, and the average flow velocity across 50 frames as μm/s. All continuous variables were tested for normality using a skewness and kurtosis and normality test. All data was normally distributed data and analyzed using paired t-test, comparing MCC under anesthetic challenge to baseline MCC. Correlations between various cilia parameters and pre-terminal MCC scan tested using the pearson correlation test. A two-tailed p-value of < 0.05 was considered significant. All analyses were performed using Stata 12.2 (StataCorp, College Station, Texas) (Fig. 5).

## Supplementary Information

Supplementary Information
