# Supplementary material for: Differential effect of anesthetics on mucociliary clearance in vivo in mice
Source: Sci Rep. 2021 Mar 1;11:4896. doi: 10.1038/s41598-021-84605-y (PMC7921682; doi:10.1038/s41598-021-84605-y)
Supplement: Supplementary file 1 — Supplementary Information [file 41598_2021_84605_MOESM1_ESM.docx]

**Differential Effect of Anesthetics on Mucociliary Clearance *in Vivo* in Mice**

Kyle S. Feldman, BS, Eunwon Kim, BS, Michael J. Czachowski, MBA, Yijen Wu, PhD,

Cecilia W. Lo, PhD, Maliha Zahid, MD, PhD

**Supplemental Material**

**Supplemental Methods**

**Analysis of Mucociliary Scans**

Process the CT and SPECT images in FIJI ImageJ^1^ using the reslice tool in to generate coronal view images from the default axial images. Then perform a z-stack sum projection on the SPECT image to add the count data from each slice and generate a single image for ease of analysis. Then resize and co-register the CT and SPECT images using the phantom tube as a reference (Supplemental Figure S3 A, B). Track and use consistent resize measurements across all samples. Then binarize the CT image using auto thresholding, followed by inverting the stack, and performing a z-stack sum projection to generate an outline of the lungs for analysis (Supplemental Figure S3 C). Then rotate the CT and SPECT images and merge the image using the channel tools. MCC can then be measured by drawing an ROI around the right lung and measuring (Supplemental Figure S3 D). This measurement will be of the total counts in the right lung for the 0h and 6h time points, which can then be corrected for radioactive decay using the formula: ${N\left( t \right)=N}_{0}e^{-t}$. ^99m^Tc-Sc has a decay constant of 3.21$e^{-5}$ per second with a half-life of ~6 hours. These values can then be used to calculate a % clearance.

**Cilia Length Analysis**

Using FIJI ImageJ, process the image using temporal median with a time window half-width of 5 and standard deviations over media for foreground setting of 3.5 to select for points of motion from the cilia (Supplemental Figure S4 A, B). Then apply a despeckling filter to replace each pixel with the median value in its 3 × 3 neighborhood (Supplemental Figure S4 C). Then perform a z-stack sum projection to combine the image data from each slice (Supplemental Figure S4 D). Perform a background subtraction to remove noise (Supplemental Figure S4 E), then process the image with a Gaussian blur filter (Supplemental Figure S4 F). The image can then be binarized after thresholding for the points of motion from the temporal median processing (Supplemental Figure S4 G). Subject the image to a series of five erode and five dilate filters to generate a binary image with masks for each spot of motion in the image, which will match with cilia. For profile view cilia, these masks provide an easy to measure shape that matches the length of the cilia (Supplemental Figure S4 H). This part of the analysis can be performed using Supplemental Macro 1.

Then use the wand tool to select a mask that outlines the cilia effectively, taking care to avoid masks that capture debris. Once a selection is made, invert the masks and subtract 255 to remove the background. Then set the image properties so that the unit is pixels, and set the width and height to 1 (Supplemental Figure S4 I). Use the thickness command of the ImageJ plugin BoneJ^2^ to measure the thickness of the mask in pixels. This measurement can then be converted into microns. This part of the analysis can be performed using Supplemental Macro 2.

**Cilia Beat Frequency Analysis**

Using FIJI ImageJ, crop an area of interest containing either single cilia or groups of cilia beating synchronously (Supplemental Figure S5 A, B). This can be done using Supplemental Macro 3. Measure the area of interest using an image calculator to measure the difference between every image in the video compared to the first image. This can be done using Supplemental Macro 4. Then graph the values and measure the average number of frames between peaks to determine beat frequency (Supplemental Figure S5 C).

**Percent Ciliation Analysis**

Using FIJI ImageJ, draw a line with the segmented line tool to along the total length of epithelia and record the measurement (Supplemental Figure S6 A). Then draw lines along the length of each ciliated section of the epithelia and record the measurements (Supplemental Figure S6 B). Percent ciliation can be calculated using the ratio of ciliated surface to total surface.

**Bead Flow Analysis**

Using FIJI ImageJ, rotate the image so that strips of motile cilia are oriented horizontally (Supplemental Figure S7 A), then crop a window along the length of the ciliated surface with the top of the window 50 pixels high (Supplemental Figure S7 B). Apply an edge detection filter (Supplemental Figure S7 C), then run a Gaussian blur filter to produce beads with defined circular shapes and low background noise (Supplemental Figure S7 D). This can be done using Supplemental Macro 5.

Analyze the image using an ImageJ plugin called Mosaic Particle Tracker, a feature point tracking algorithm that can be used to automatically track and quantify particle trajectories in videos (Supplemental Figure S7 E). This tracker utilizes inputted values for approximate particle radius, a cutoff score for non-particle discrimination, an intensity threshold percentile for particle discrimination, a maximum pixel displacement value for movement between frames, and a link range for the number of future frames to consider when linking particles between frames. When the analysis is performed, the tracker can output x and y coordinates for each particle tracked^3^. Particle tracks greater than 50 frames were determined to be real tracks as opposed to background noise. Particle tracks greater than 300 frames were determined to be tracks of large, immobile objects instead of beads. Particle tracks within the range 50-300 frames were analyzed.

**Supplemental Results**

**Supplemental Figure S1.** Individual clearances for 8-week old C57BL/6J mice. This data was generated from an original set of nine mice for the purpose of identifying which mice had large enough baseline clearances for use in the study. The five mice with the highest MCC were treated with anesthetics while the remaining four mice were excluded from further treatments.

**Supplemental Figure S2.** SPECT/CT images of an MCC scan. A) A SPECT image that has been co-localized with a CT image. B) A CT image with a visible phantom tube that was used for co-localization. C) A mask of the airway derived by binarizing the CT image and performing a z-stack sum projection. D) The CT mask co-localized with the SPECT image. An ROI for analysis has been drawn around the right lung.


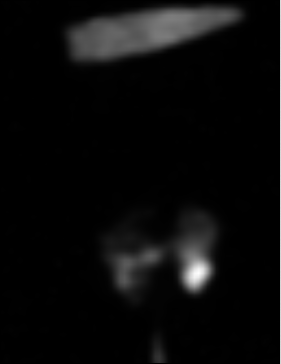

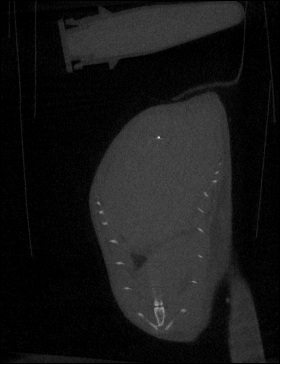

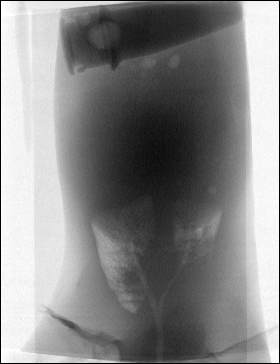

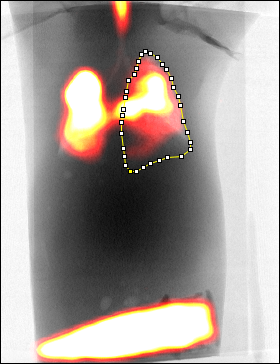


A

B

C

D

**Supplemental Figure S3.** A) Airway video-microscopy image used to analyze cilia length. B) The image was processed using temporal median to detect motion. C) A despeckling filter was applied to remove background. D) A z-stack sum projection was performed. E) A background subtraction was performed to remove more noise. F) A gaussian blur filter was applied. G) The image was binarized by thresholding for motion points. H) Filters were applied to emphasize bulk motion and suppress noise. I) A shape was selected for analysis using the wand tool and isolated.


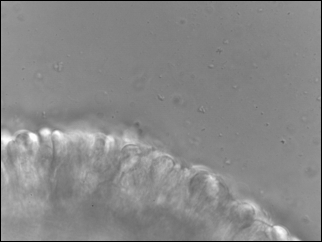

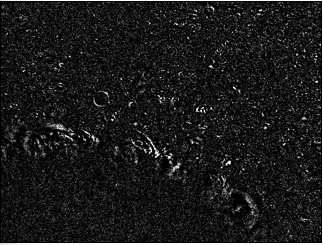

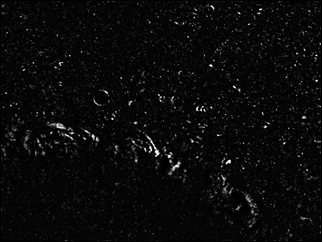

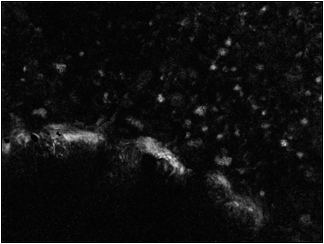

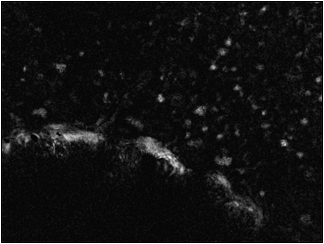

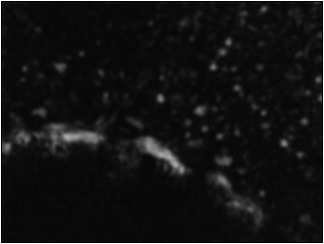

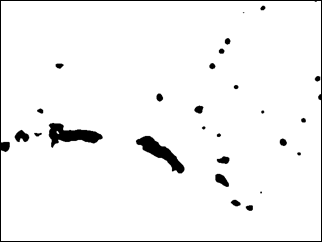

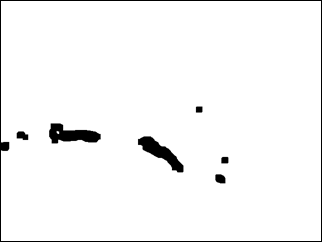

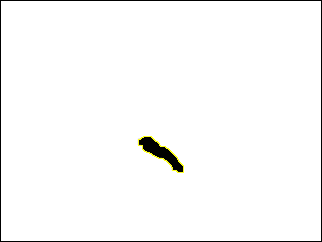


A

B

C

D

E

F

G

H

I

**Supplemental Figure S4.** A) An ROI was drawn to capture a group of synchronously beating cilia. B) The ROI enlarged. C) A graph of difference over time that can be used to measure the number of frames between peaks to determine beat frequency.


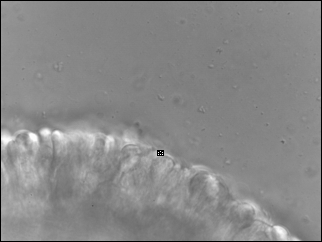

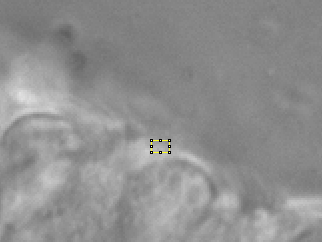


A

B

C

**Supplemental Figure S5.** A) A line was drawn along the total length of the epithelia. B) Lines were drawn along the length of every ciliated section.


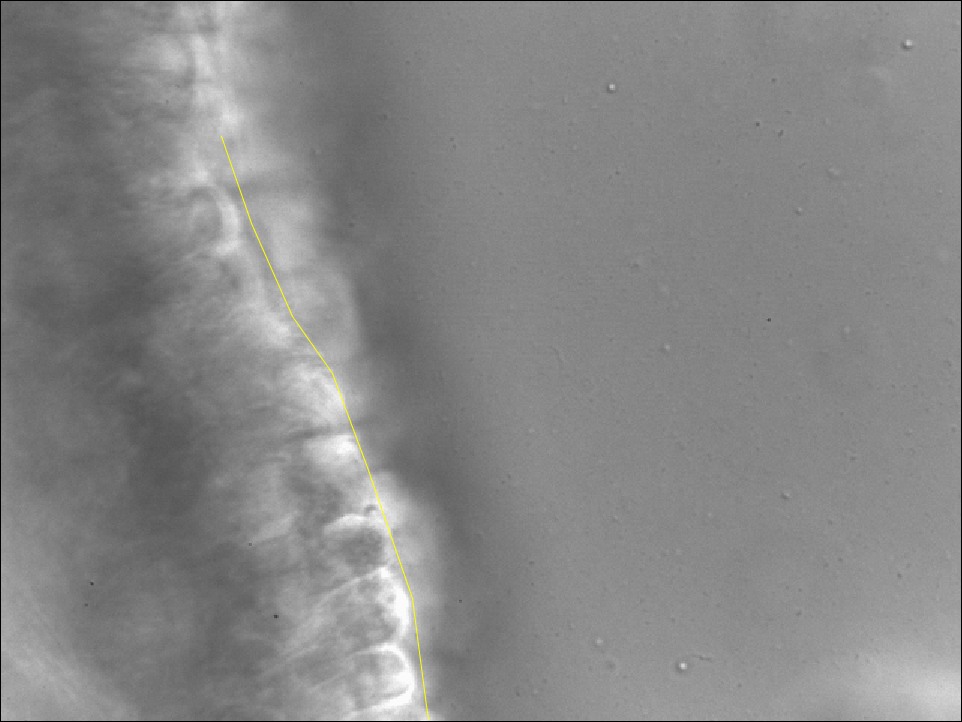

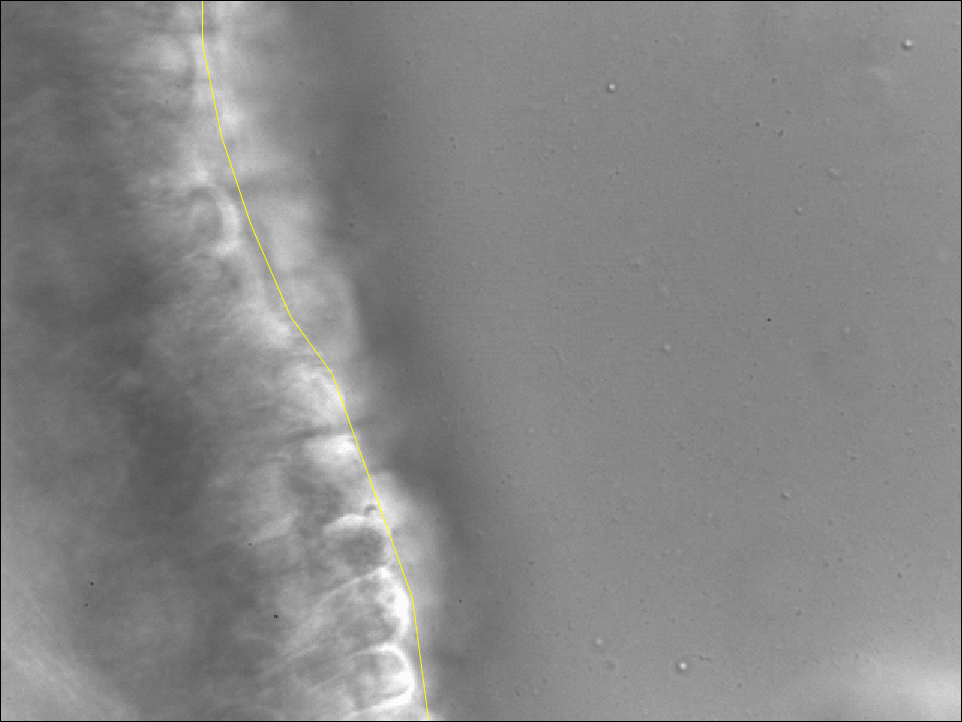


A

B

**Supplemental Figure S6.** A) A video microscopy image of ciliated airway epithelia. B) The image was rotated and an ROI 50 pixels high was drawn above the cilia. C) An edge detection filter was applied. D) A gaussian blur filter was applied. E) The bead tracking analysis was performed.


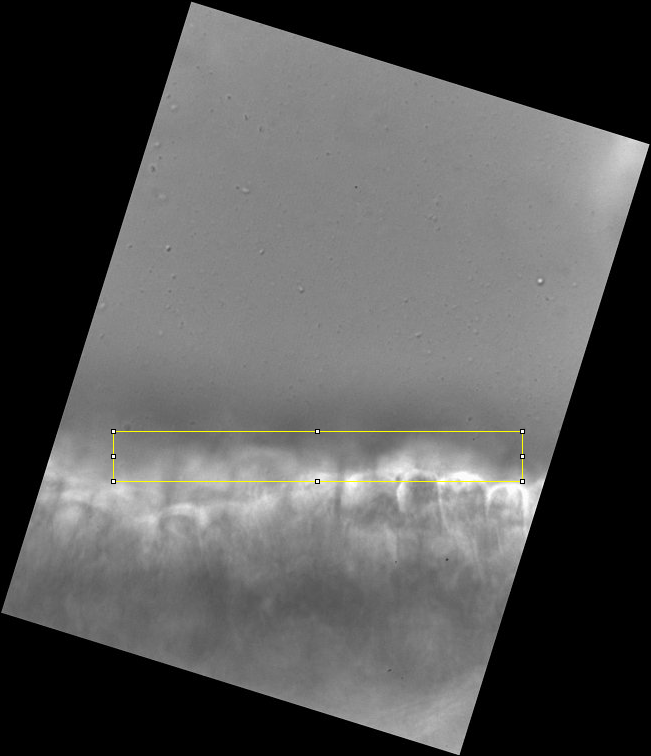

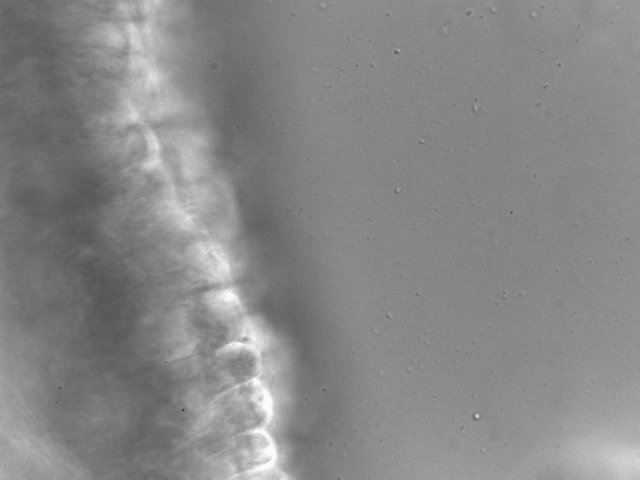

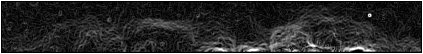

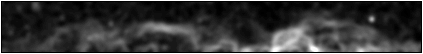

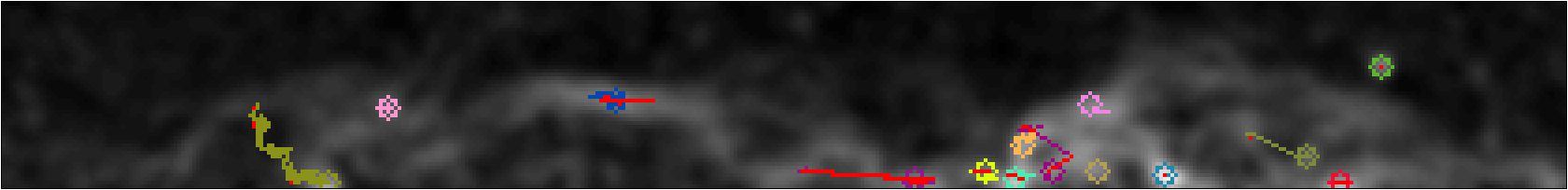


A

B

C

D

E

**Supplemental Table S1**

| **Groups** | **Mean C-P Ratio** | **Std. Dev C-P ratio** | **p-value** |
| --- | --- | --- | --- |
| Baseline | 1.9 | 0.8 |  |
| Iso | 1.5 | 0.4 | 0.2805 |
| Ket/Xyl | 1.7 | 1.1 | 0.7199 |
| Dex/Ket | 1.6 | 0.7 | 0.5489 |
| Fen/Iso | 1.9 | 0.7 | 0.9036 |
| Propofol | 2.2 | 0.4 | 0.3096 |
| Mid/Dex/Fen | 2.2 | 0.8 | 0.4445 |

**Supplemental Code**

**Supplemental Macro 1:**

files = getDirectory("Movies to process");

list = getFileList(files);

n = lengthOf(list);

count=0;

maskDir = files+"/masks/"

if(!File.exists(maskDir)) {

File.makeDirectory(maskDir);

}

Dialog.create("Sensitivity Options");

Dialog.addNumber("threshold:", 3.68);

Dialog.show();

threshold = Dialog.getNumber();

setBatchMode(true);

for(i=0;i<n;i++) {

open(files+list[count]);

name=getTitle();

run("Slice Keeper", "first=1 last=400 increment=6");

run("Temporal Median", "5, 3.5");

run("Despeckle", "stack");

run("Z Project...", "projection=[Sum Slices]");

run("Subtract Background...", "rolling=50");

run("Gaussian Blur...", "sigma=3");

setThreshold(threshold, 30);

run("Make Binary");

run("Erode");

run("Erode");

run("Erode");

run("Erode");

run("Erode");

run("Dilate");

run("Dilate");

run("Dilate");

run("Dilate");

run("Dilate");

maskName = getTitle();

run("Add Slice");

selectWindow(name);

run("Copy");

selectWindow(maskName);

run("Paste");

saveAs("Tiff", maskDir+name+"_mask");

close();

close();

close();

close();

count++;

}

**Supplemental Macro 2:**

files = getDirectory("Masks to analyze");

list = getFileList(files);

n = lengthOf(list);

count=0;

setBatchMode(false);

for(i=0;i<n;i++) {

open(files+list[count]);

x=getWidth();

y=getHeight();

maskName = getTitle();

selectWindow(maskName);

Stack.setSlice(2);

run("Delete Slice");

setTool("Wand");

waitForUser("Choose the cilia, Shift+click OK to skip");

if (isKeyDown("Shift") == true) {

selectWindow(maskName);

run("Close");

}

else {

run("Make Inverse");

run("Subtract...", "value=255 stack");

run("Select None");

run("Properties...", "unit=pixel pixel_width=1 pixel_height=1");

run("Thickness", "thickness graphic mask");

processed = getTitle();

selectWindow("Results");

saveAs("Results", files+"Cilia_length.xls");

selectWindow(maskName);

run("Close");

selectWindow(processed);

run("Close");

count++;

}

}

**Supplemental Macro 3:**

setBatchMode(false);

files = getDirectory("Raw movies");

list = getFileList(files);

n = lengthOf(list);

output = files + "/_cropped/"

File.makeDirectory(output);

count = 0;

result = nResults();

for (i=0; i < n; i++) {

open(files+list[i]);

stack = getTitle();

setTool("rectangle");

waitForUser("Select the ROI, or Shift+OK to skip");

if (isKeyDown("Shift") == true) {

selectWindow(stack);

run("Close");

}

else {

run("Crop");

saveAs("Tiff", output+stack);

new = getTitle();

selectWindow(new);

run("Close");

}

count++;

}

print("Finished");

**Supplemental Macro 4:**

setBatchMode(true);

if(isOpen("Results")) {

selectWindow("Results");

run("Close");

}

run("Clear Results");

setResult("Title", 0, 1);

run("Set Measurements...", "mean redirect=None decimal=5");

files = getDirectory("Movies");

count = 0;

list = getFileList(files);

n = lengthOf(list);

for (i=0; i < n; i++) {

open(files+list[count]);

stack = getTitle();

fileName = list[i];

slices = nSlices();

run("Gaussian Blur 3D...", "x=1 y=1 z=1");

selectWindow(stack);

setSlice(1);

run("Duplicate...", "use");

first = getTitle();

imageCalculator("Difference create stack", stack, first);

diff = getTitle();

for (slice=1; slice<=nSlices-1; slice++){

selectWindow(diff);

setSlice(slice+0);

run("Measure");

number = (nResults-1);

setResult("Title", number, stack);

}

selectWindow(stack);

run("Close");

selectWindow(diff);

run("Close");

selectWindow(first);

run("Close");

selectWindow("Results");

saveAs("Results", files+fileName+"Cilia_Beat.xls");

run("Close");

count++;

}

**Supplemental Macro 5:**

originalTitle = getTitle();

setTool("line");

waitForUser("Draw rotation line, or Shift+OK to skip");

if (isKeyDown("Shift") == true) {

selectWindow(stack);

run("Close");

}

else {

run("Clear Results");

run("Measure");

Angle = getResult("Angle",0);

run("Duplicate...", "duplicate");

run("Rotate... ", "angle=Angle grid=1 interpolation=Bicubic stack enlarge");

run("Clear Results");

setTool("rectangle");

waitForUser("Select the ROI, or Shift+OK to skip");

if (isKeyDown("Shift") == true) {

selectWindow(stack);

run("Close");

}

else {

run("Crop");

cropTitle = getTitle();

run("8-bit");

run("16-bit");

run("Find Edges", "stack");

run("Gaussian Blur...", "sigma=2 stack");

setTool("line");

rename(originalTitle);

run("Enhance Contrast", "saturated=0.35");

}

}

**Supplemental References**

1. Schindelin J, Arganda-Carreras I, Frise E, Kaynig V, Longair M, Pietzsch T, Preibisch S, Rueden C, Saalfeld S, Schmid B, Tinevez J-Y, White DJ, Hartenstein V, Eliceiri K, Tomancak P, Cardona A: Fiji: an open-source platform for biological-image analysis. Nat Methods 2012; 9:676

2. Doube M, Kłosowski MM, Arganda-Carreras I, Cordelières FP, Dougherty RP, Jackson JS, Schmid B, Hutchinson JR, Shefelbine SJ: BoneJ: Free and extensible bone image analysis in ImageJ. Bone 2010; 47:1076–9

3. Sbalzarini IF, Koumoutsakos P: Feature point tracking and trajectory analysis for video imaging in cell biology. J Struct Biol 2005; 151:182–95
